# Mobile phone short message service (SMS) as a malaria control tool: a quasi-experimental study

**DOI:** 10.1186/s12889-019-7336-6

**Published:** 2019-08-29

**Authors:** Aliyu Mohammed, Princess Ruhama Acheampong, Easmon Otupiri, Francis Adjei Osei, Roderick Larson-Reindorf, Ellis Owusu-Dabo

**Affiliations:** 1 School of Public Health, Kwame Nkrumah University of Science and Technology (KNUST), Kumasi, Ghana; 2 Public Health Unit, Komfo-Anokye Teaching Hospital, Kumasi, Ghana; 3 Obstetrics and Gynaecology Department, Komfo Anokye Teaching Hospital, Kumasi, Ghana

**Keywords:** mHealth, Mobile phone, Malaria, Behavior change communication, Child health, Extended parallel process model (EPPM)

## Abstract

**Background:**

Despite the extensive implementation of control measures and achievements in morbidity reductions, malaria continues to contribute to substantial morbidity and mortality in children under-five. Innovative approaches involving the use of mobile phones have been suggested to improve health outcomes. However, evidence of its effect on reducing the prevalence of malaria is limited. This study, therefore, aimed to assess the effect of a theory-driven mHealth intervention on the prevalence of malaria among children under-five living in rural districts of Ghana.

**Methods:**

We conducted a quasi-experimental study of a 12-month intervention using a random sample of 332 caregivers with children under-five from two rural districts, assigned to either an intervention or a control group. Caregivers in the intervention group received voice short message service (SMS) on malaria prevention based on a behavior change theory to improve their health behaviors and practice, once a week for twelve months, while caregivers in the control group received none. Pre- and post-intervention assessment of the treatment effect (ATT) on malaria in children under-five was conducted using propensity score and difference-in-difference (DiD) analyses.

**Results:**

Among children whose caregivers received the intervention, the prevalence of malaria decreased from 58.4% at baseline to 37.8% at endline (difference: -20.6%; 95% CI: − 31.1, − 10.1) compared with children in the control group, where a reduction of 65.0 to 59.9% (difference − 5.1%; 95% CI: − 15.5, 5.4) was observed. The treatment effect at endline revealed a statistically significant reduction in malaria prevalence (ATT: -0.214; 95% CI: − 0.36, − 0.07) compared with the baseline (ATT: -0.035; 95% CI: − 0.16, 0.09). Overall, the intervention effect showed a significant reduction in the prevalence of malaria among children under-five was positive (DiD: − 0.154; *p* = 0.043).

**Conclusion:**

The results of the study indicate the effectiveness of mobile phone SMS as a control tool for reducing the burden of malaria in children under-five.

**Electronic supplementary material:**

The online version of this article (10.1186/s12889-019-7336-6) contains supplementary material, which is available to authorized users.

## Background

Worldwide, millions of children under-five continue to die before their fifth birthday, with almost all of such deaths occurring in developing countries; sub-Saharan Africa alone accounts for almost half of these deaths [1]. Malaria together with pneumonia and diarrhoea remain among the leading causes of the deaths – accounting for almost one-third of the global under-five deaths, and about 40% of under-five deaths in sub-Saharan Africa [2].

Malaria is endemic in Ghana and continues to pose a public health threat, especially to children under-five living in the rural parts of the country with limited access to health care. The 2015–2020 Ghana Malaria Strategic Plan aims to reduce malaria burden by 75.0% by the year 2020 [3]**.** As part of the efforts to control malaria in the country, a number of control programs including the Roll Back Malaria Initiative and Home Management of Malaria were initiated to implement a combination of curative and preventive interventions. The main activities of the malaria control programme in Ghana include the distribution of insecticide-treated bed nets at health facilities and to households with pregnant women and children under-five, intermittent preventive treatment for infants, intermittent preventive treatment in pregnancy, *indoor residual spraying,* seasonal malaria *chemoprevention, and social behavior change communication*. Unfortunately, these control measures undertaken over the years have been met with limited success due to poor health infrastructure, focus on single strategies, lack of funding, poor human resource capacity and non-involvement of some stakeholders [4].

An innovative approach involving the use of mobile phones is yet to be explored in Ghana as a potential malaria control tool. Mobile health (mHealth), involving the use of mobile devices to support clinical and public health care has the potential to improve the numerous healthcare challenges faced by many developing countries [5]. For example, studies on mHealth have reported improvement in vaccination coverage [6], disease and treatment monitoring of malaria [7], and management of some chronic diseases including diabetes and HIV [8–10]. With over 30 million mobile subscriptions and a penetration rate of 119% in Ghana [11], the ubiquity and the multi-purpose function of a mobile phone, which allows for short message service (SMS), presents an unparalleled opportunity for disease prevention and control efforts. SMS is a component of mobile device systems which uses communication protocols standardized in the Global System for Mobile communications, allowing messages to be interchanged from a mobile phone or a computer to one or many mobile phones simultaneously [12]. Most mHealth interventions involving the use of SMS in Africa have been assessed in the area of management of chronic diseases and long-term therapy [13], with little done in the area of acute diseases such as malaria [14]. Mobile phone text messaging, in particular, has been shown to be an effective means for improving health service delivery in diabetes case management [15], smoking cessation support [16] and weight loss as well as for improving behavioral change outcomes [17].

In order to prevent early childhood death and improve survival, caregivers must change their health behaviors. A growing body of evidence suggests that public health and health-promotion interventions that are based on social and behavioral science theories are more effective than those lacking a theoretical basis [18–20]. Social Behavior Change Communication (SBCC) interventions, involving the strategic use of communication to promote positive health outcomes based on proven theories and models of behavior change form a key part of all types of health promotion and disease prevention. Previous studies on SBCC suggest that exposure to ‘threat’ and ‘efficacy’ messages are associated with preventive behaviors [21–23]. Our mHealth intervention study relied exclusively on the Extended Parallel Process Model (EPPM) of behavior change [24]. This fear appeal model holds the view that the effectiveness of a fear appeal message depends on the extent of the perceived threat of an event relative to the perceived efficacy of an individual. Thus, positive changes in attitudes, behaviors, and intentions could be observed if beliefs about an individual’s ability to perform the recommended response to avert the threat (self-efficacy) and beliefs about the effectiveness of the recommended response in deterring or avoiding the threat (response efficacy), exceeds one’s beliefs about the significance or magnitude of the threat (perceived severity) and beliefs about one’s risk of experiencing the threat (perceived susceptibility).

Despite the potential of mHealth, little is known about its effect on the prevalence of malaria among children under-five living in rural settings with limited access to health care. We hypothesised that this theory-driven mHealth intervention could significantly lead to a reduction in the prevalence of malaria among children whose caregivers received the mHealth intervention compared with those who did not. The objective of this study, therefore, was to examine the effect of the theory-driven mHealth intervention, delivered by one-way voice SMS approach to caregivers, on the prevalence of malaria among children under-five living in a rural district of Ghana.

## Methods

### Study design and sites

We conducted a quasi-experimental study, using pre- and post-intervention surveys, in a rural district of the Asante Akim North District (6°30′ - 7°30′N, 0°15′ - 1° 20′W), located in the Ashanti Region of Ghana from February 2016 to March 2017. The district has four sub-districts, of which two were randomly selected; one as the intervention area (Agogo) and another as the control area (Juansa). The two study areas are approximately thirteen (13) kilometres apart, which helped to reduce any possible contamination. Both areas are dominated by the Akan ethnicity, with subsistence farming as their main source of income. The region is characterized by high malaria transmission especially during the rainy season from May–July through to August [25]. The prevalence of malaria as measured by Rapid Diagnostic Test (RDT) among children 6–59 months according to the 2014 Ghana Demographic and Health Survey (GDHS) was 20.6% [25]. The annual entomological inoculation rates (AEIR) of some areas of the district range from 40 to 158 for the predominant species of *Anopheles* mosquito (*Anopheles gambiae* and *Anopheles funestus*) [26]. The district has nine (9) health facilities; four (4) in the intervention area and three (3) in the control area. Mobile telecommunication networks operating in the district included Vodafone, Tigo, Mobile Telephone Networks (MTN) and Airtel.

### Study population

We included primary caregivers and their children under-five. A primary caregiver was defined in this study as a person who takes primary responsibility and is the legal guardian of the child under-five. We only included caregivers who were able to give written informed consent and had access to a mobile phone – either owned by themselves or their families but accessible to the caregiver. The intervention was designed to target the health behaviors of the caregivers.

### The intervention

Telephone numbers obtained from caregivers during recruitment were uploaded onto a mobile phone-based health information system (mHIS). This system was setup to automatically deliver a one-way voice SMS to only the intervention group at their own convenient time – received in the form of a phone call. Scheduled based on the time indicated by the caregivers, the health messages were delivered automatically once every two weeks to the caregivers via their mobile phones in their local language (Twi) for twelve (12) months. The content of the message, which was designed to improve the health behaviors of the caregivers was developed based on the EPPM of behavior change. The content of the message was motivational, designed to prompt the caregiver and provide cues for action on how to protect their children from malaria. An example of the messages was, “Malaria can kill your child if you don’t protect them by consistently using an insecticide-treated net.” In order to maintain the interest of the caregivers, the content of the messages was varied on 5 themes (see Additional file 1). The messages were alternated for better understanding of the themes and for recollection. The messages were sent in bulk and tailored to suit the time indicated by the caregiver during recruitment. The messages were automatically re-sent to caregivers who did not receive them, repeated three (3) times with an hour interval in a day for three (3) days. In total, 12, 064 voice SMS were successfully delivered to caregivers in the intervention group over the 12-month intervention period.

In the control group, participants did not receive any SMS, but were interviewed at baseline and endline.

### Sampling and sample size

We conducted a cross-sectional baseline and endline surveys to assess the prevalence of malaria among children under-five in both the intervention and control sites by employing a two-stage random cluster sampling method. Our study was designed to detect a 15% reduction in the prevalence of malaria in the intervention arm over the 12-month period of the intervention, based on outcome improvements observed in other studies that employed the use of SMS [6, 27]. The sample size was calculated using Stata version 14. The study had 80% statistical power and an alpha error set at α = 0.05, using a two-tailed chi-square test, and a 21% malaria prevalence in the study area [25]. The minimum sample size required in each arm was 138. However, factoring in attrition of 20%, a total sample of 333 caregivers with children under-five, 166 in each arm, was sufficient to detect a significant difference between the intervention and control groups.

Ten (10) participants dropped out of the study due to personal reasons, giving a response rate of 97%; 97.5% for the intervention group and 98.8% for the control group. We first selected the community clusters within each study area and then selected households in the study communities. Based on the World Health Organization (WHO) cluster-sampling methodology, we randomly selected 20 clusters (communities) from each study site. In each cluster, we listed all households with children under-five and randomly selected an average of 8 households per cluster, representing approximately 20% of the households. Where a household had more than one eligible caregiver and child, we randomly selected one of each for the survey. A household was defined as a group of people living in the same dwelling space and who acknowledged the authority of a man or woman whom they mostly depend on for their livelihoods.

### Data collection

Caregivers who consented to participate in the study were recruited and registered. The registration involved recording of their names, mobile phone numbers, the name of community and Global Positioning System (GPS) data, and consequently exported to the mHIS database. Using a structured questionnaire, we collected information on the age and sex of all children under-five. In addition, caregivers’ demographic characteristics such as age, education level, marital status, religion, parity, access to and use of mobile phones were recorded. We assessed the primary endpoint, prevalence of malaria at baseline and endline, by asking the caregivers whether the child had experienced fever in the last twelve (12) months preceding the survey. Information on all children under-five included in the study who were taken to a health facility was retrieved from the health records and recorded and checked for the diagnosis: malaria was confirmed by either Rapid Diagnostic Test (RDT) or microscopy. For children who were treated at home, further questions were asked to ascertain whether the child was provided with an anti-malaria drug. An episode of malaria in our study was defined as a child having a fever and being confirmed by either RDT or microscopy, or being treated with anti-malaria at home by the caregiver based on the signs shown by a febrile child. The secondary endpoint, behavior change, was assessed using a Likert-scale responses [24, 28, 29], ranging from 1 (*strongly disagree*) to 5 (*strongly agree*). Questions measuring the key aspects of the behavior change theory such as perceived severity, susceptibility, self-efficacy and response efficacy were assessed at baseline and repeated at endline. Table 1 shows the internal consistency of the constructs, generated using the composite score of the items.

**Table 1:** Internal consistency of the EPPM constructs

Indicator	Number of items	Cronbach’s alpha (α)	
		Before	After
Threat			
Severity	3	0.52	0.68
Susceptibility	5	0.68	0.87
Efficacy			
Self-efficacy	6	0.62	0.91
Response efficacy	4	0.85	0.90

**Fig. 1:**
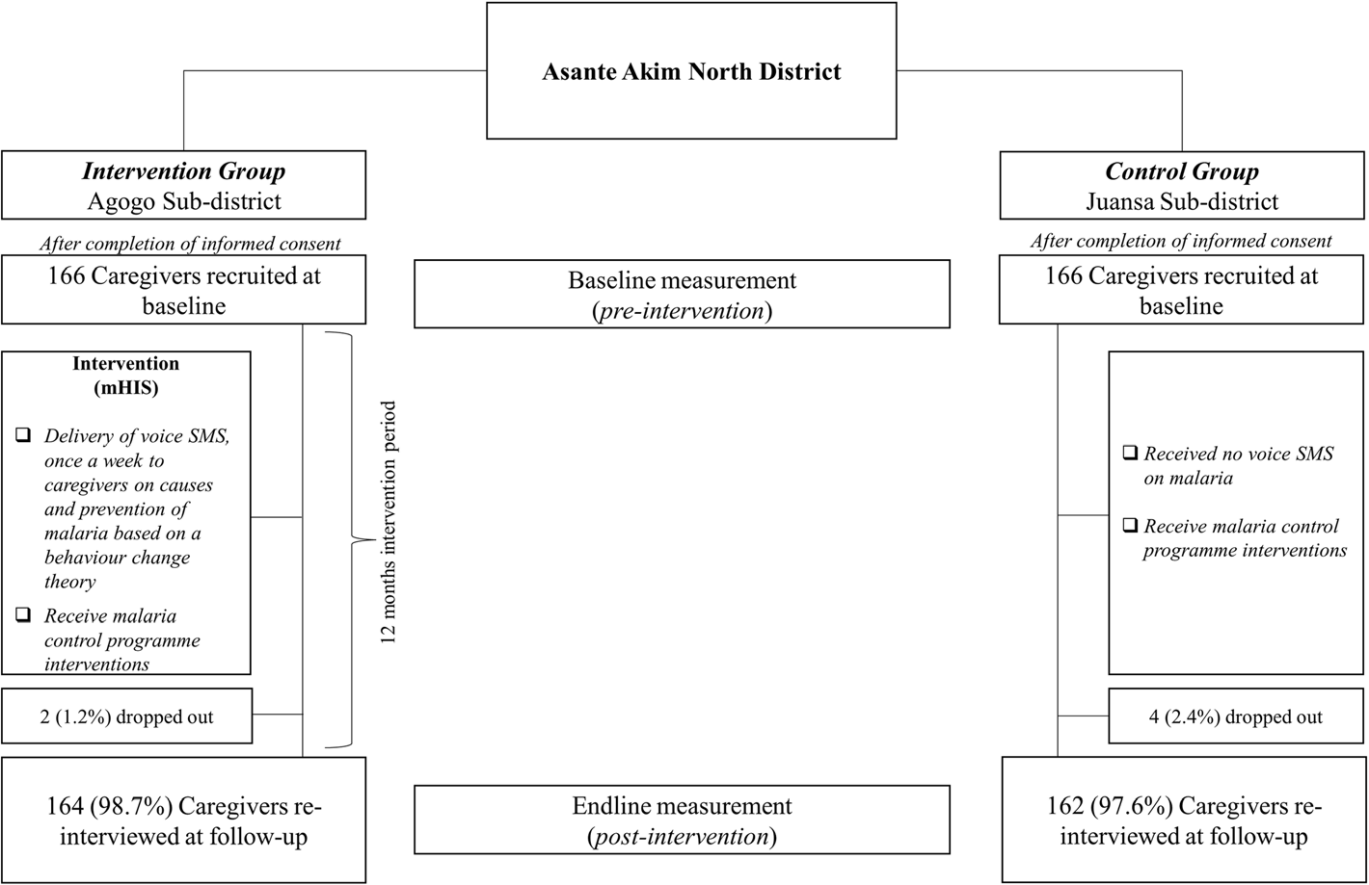
Recruitment and follow-up of study participants

The pre-intervention questionnaire was administered during early February 2016 and respondents were followed up after the 12 months intervention period as shown in Fig. 1.

### Data analysis

We used Chi-square/Fisher’s exact test to examine the pre-existing differences between the intervention and control group with regard to the baseline sociodemographic characteristics of the caregivers and the children, with *p*-values < 0.05 considered statistically significant. Malaria prevalence was compared between baseline and endline surveys using t-test. This was done in two stages; first, confirmed and presumptive cases and only confirmed cases for the second stage.

We conducted Propensity Score Matching (PSM) analysis to obtain approximately unbiased estimates of the Average Treatment Effect on the Treated (ATT) by improving covariate balance. The greedy nearest neighbor method was used to estimate the ATT after estimating the propensity scores for each participant. *Sensitivity analysis for matching estimators* [30] was conducted to simulate a potential confounder (“read-write” variable) in order to assess the robustness of the estimated treatment effect with respect to deviations from the Conditional Independence Assumption (CIA). The difference-in-differences (DiD) model was used to examine the differences in malaria prevalence from baseline to endline between the intervention and control group. The DiD approach was used to account for the trends in the outcome and unobserved time-invariant differences between the intervention and control groups, providing an unbiased estimate of the intervention effect. We included variables such as the age of caregiver, marital status and wealth as confounding variables to control for the pre-existing varying population composition between the two groups. Stata 14 (Stata Corp., College Station, Texas, United States) was used for the analysis.

## Results

### Study participants

The socio-demographic characteristics of the study sample at baseline are shown in Table 2. We sampled a total of 332 caregivers and a proportional number of children under-five for this study; 6 were lost to follow-up. The mean age of the children was 2.7 years (SD ± 1.1), with more than half of them being male (52.1%). The mean age of the caregivers was 31.4 years (SD ± 9.2), 54.5% married and 61.1% had completed Middle level or Junior High School (JHS). The Chi-square (*χ*^2^)/Fisher’s exact tests revealed a statistically significant difference between the two groups with respect to the sex of the child (*p* < 0.001), marital status of the caregiver (*p* < 0.001) and household wealth (*p* = 0.006) Table 2.

**Table 2:** Sociodemographic characteristics of children and caregivers in intervention and control areas

Variable	Total n (%)	Intervention	Control	*χ*^2^ (*p-value*)
		n (%)	n (%)	
Child characteristics:				
Age (years)				0.717
≤ 1	60 (18.1)	33 (19.9)	27 (16.3)	
2	64 (19.3)	29 (17.5)	35 (21.1)	
3	135 (40.7)	69 (41.6)	66 (39.8)	
4	48 (14.5)	23 (13.9)	25 (15.1)	
Total	332 (100.0)	166 (100.0)	166 (100.0)	
Mean age (SD)	2.7 (1.1)	2.7 (1.1)	2.8 (1.1)	
Sex				<0.001
Male	173 (52.1)	70 (42.2)	103 (62.0)	
Female	159 (47.9)	96 (57.8)	63 (38.0)	
Total	332 (100.0)	166 (100.0)	166 (100.0)	
Caregiver characteristics:				
Age (years)				0.149
<20	27 (8.1)	9 (5.4)	18 (10.8)	
21 – 30	152 (45.8)	79 (47.6)	73 (44.0)	
31 – 40	112 (33.7)	61 (36.8)	51 (30.7)	
41+	41 (12.4)	17 (10.2)	24 (14.5)	
Total	332 (100.0)	166 (100.0)	166 (100.0)	
Mean age (SD)	31.4 (9.2)	31.2 (7.9)	31.6 (10.2)	
Marital status				<0.001
Divorced	17 (5.2)	5 (3.1)	12 (7.2)	
Separated	36 (10.8)	26 (15.6)	10 (6.0)	
Married	181 (54.5)	99 (59.6)	82 (59.6)	
Never married	51 (30.7)	21 (12.7)	51 (30.7)	
Cohabitation	26 (7.8)	15 (9.0)	11 (6.3)	
Total	332 (100.0)	166 (100.0)	166 (100.0)	
Religion				0.817
Christian	308 (93.0)	155 (93.4)	153 (92.6)	
Muslim	23 (7.0)	11 (6.6)	12 (7.3)	
Total	332 (100.0)	166 (100.0)	166 (100.0)	
Education level^†^				0.668
No formal education	26 (7.8)	11 (6.6)	15 (9.0)	
Primary	54 (16.3)	31 (18.7)	23 (1.9)	
Middle/ JHS	203 (61.1)	100 (60.2)	103 (62.1)	
Secondary	41 (12.4)	21 (12.7)	20 (12.1)	
Tertiary	8 (2.4)	3 (1.8)	5 (3.0)	
Total	332 (100.0)	166 (100.0)	166 (100.0)	
Household Wealth Index				0.006
Quintile 1(lowest)	72 (21.7)	46 (27.7)	26 (15.7)	
Quintile 2	74 (22.3)	39 (23.5)	35 (21.1)	
Quintile 3	63 (19.0)	35 (21.1)	28 (16.9)	
Quintile 4	60 (18.1)	21 (12.7)	39 (23.5)	
Quintile 5	63 (19.0)	25 (15.1)	38 (22.9)	
Total	332 (100.0)	166 (100.0)	166 (100.0)	

^†^Estimated using Fisher’s exact test

**Fig. 2:**
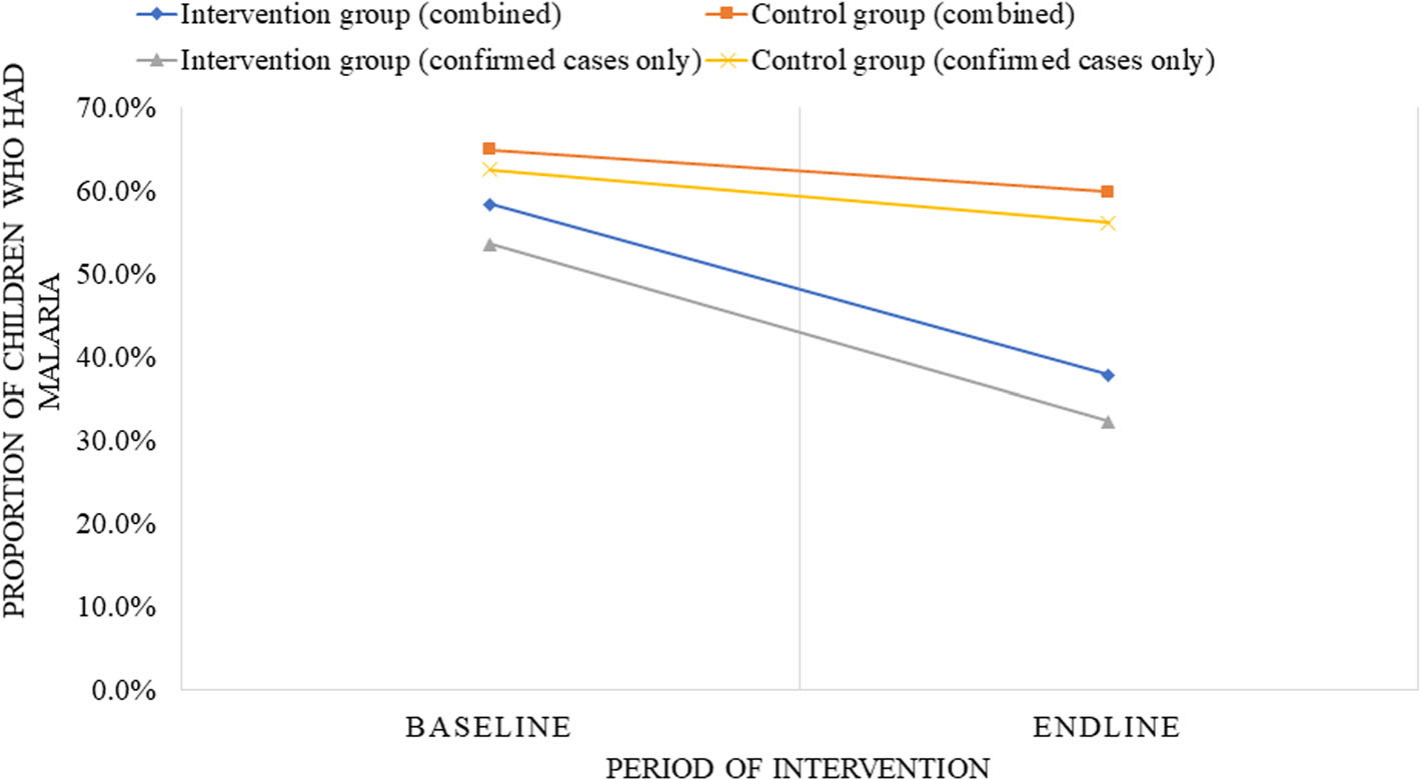
Change in the prevalence of malaria before and after the intervention

We observed a higher reduction in the prevalence of malaria among children under-five in the intervention group compared with the control group at endline (Fig. 2). The prevalence of malaria decreased from 58.4% at baseline to 37.8% at endline (difference: − 20.6%; 95% CI: − 31.1, − 10.1) compared with the control group, where a reduction of 65.0 to 59.9% (difference: − 5.1%; 95% CI: − 15.5, 5.4) was observed when confirmed and presumptive cases were considered together (Table 3). Similarly, there was a reduction in the prevalence from 53.6% at baseline to 32.3% at endline (difference: − 21.3%; 95% CI: − 31.7, − 10.9) for the intervention group compared with the control group, where a reduction of 62.6 to 56.2% (difference: − 6.4%; 95% CI: − 17.1, 4.1) was observed when only confirmed cased were considered (Table 4).

**Table 3:** Prevalence of malaria in children under-five before and after the intervention (*confirmed and presumptive cases*)

Indicator	Intervention (Agogo)			Control (Juansa)		
	Baseline n (%)	Endline n (%)	Difference (95% CI)	Baseline n (%)	Endline n (%)	Difference (95% CI)
Malaria			-20.6** (−32.6, −11.6)			−5.1 (− 15.5, 5.4)
Yes	97 (58.4)	62 (37.8)		108 (65.0)	97 (59.9)	
No	69 (41.6)	102 (62.2)		58 (34.9)	65 (40.1)	
Total	166 (100.0)	164 (100.0)		166 (100.0)	162 (100.0)	

**p < 0.001

**Table 4:** Prevalence of malaria in children under-five before and after the intervention (*Confirmed cases only*)

Indicator	Intervention (*Agogo*)			Control (*Juansa*)		
	Baseline n (%)	Endline n (%)	Difference (95% CI)	Baseline n (%)	Endline n (%)	Difference (95% CI)
Malaria			−21.3*** (−31.7, −10.9)			−6.4 (−17.1, 4.1)
Yes	89 (53.6)	53 (32.3)		104 (62.6)	91 (56.2)	
No	77 (46.4)	111 (67.7)		62 (37.3)	71 (43.8)	
Total	166 (100.0)	164 (100.0)		166 (100.0)	162 (100.0)	

***p < 0.001

### Behavior change

Table 5 shows the results of the secondary endpoints, which compares the intervention and control groups with respect to behavior change outcomes. Paired *t*-tests were undertaken to examine the change in perceptions of threat and efficacy towards malaria. According to the behavior change model, EPPM, comparison of these perceptions allows determination of whether people would engage in danger control and avert the threat or not. With the exception of response efficacy, the intervention group had a significant improvement in perceived severity (difference: + 0.35; *p* < 0.01), perceived susceptibility (difference: 0.25; *p* < 0.01) and self-efficacy (difference: + 0.35; *p* < 0.001) at post intervention. In contrast, there were significant reductions in these perceptions among the control group at post intervention.

**Table 5:** Comparison of the behavior change model (EPPM) constructs between the intervention and control group at baseline and endline

EPPM Constructs	Intervention group			Control group		
	Baseline *n* = 166	Endline *n* = 164	Diffience	Baseline *n* = 166	Endline *n* = 162	Difference
	Mean (SD)	Mean (SD)	Mean	Mean (SD)	Mean (SD)	Mean
Threat						
Perceived severity	3.55 (1.11)	3.90 (0.76)	+ 0.35**	4.07	3.53	−0.54***
Perceived susceptibility	4.15 (0.91)	4.4 (0.60)	+ 0.25**	4.4	3.83	−0.57***
Efficacy						
Response efficacy	4.00 (1.19)	3.99 (0.94)	−0.01	4.04	3.93	−0.11
Self-efficacy	4.00 (1.00)	4.36 (0.65)	+ 0.36***	4.51	3.91	−0.60***

*p < 0.05

**p < 0.01

***p < 0.001

*SD* = Standard deviation

### Treatment effect

**Fig. 3:**
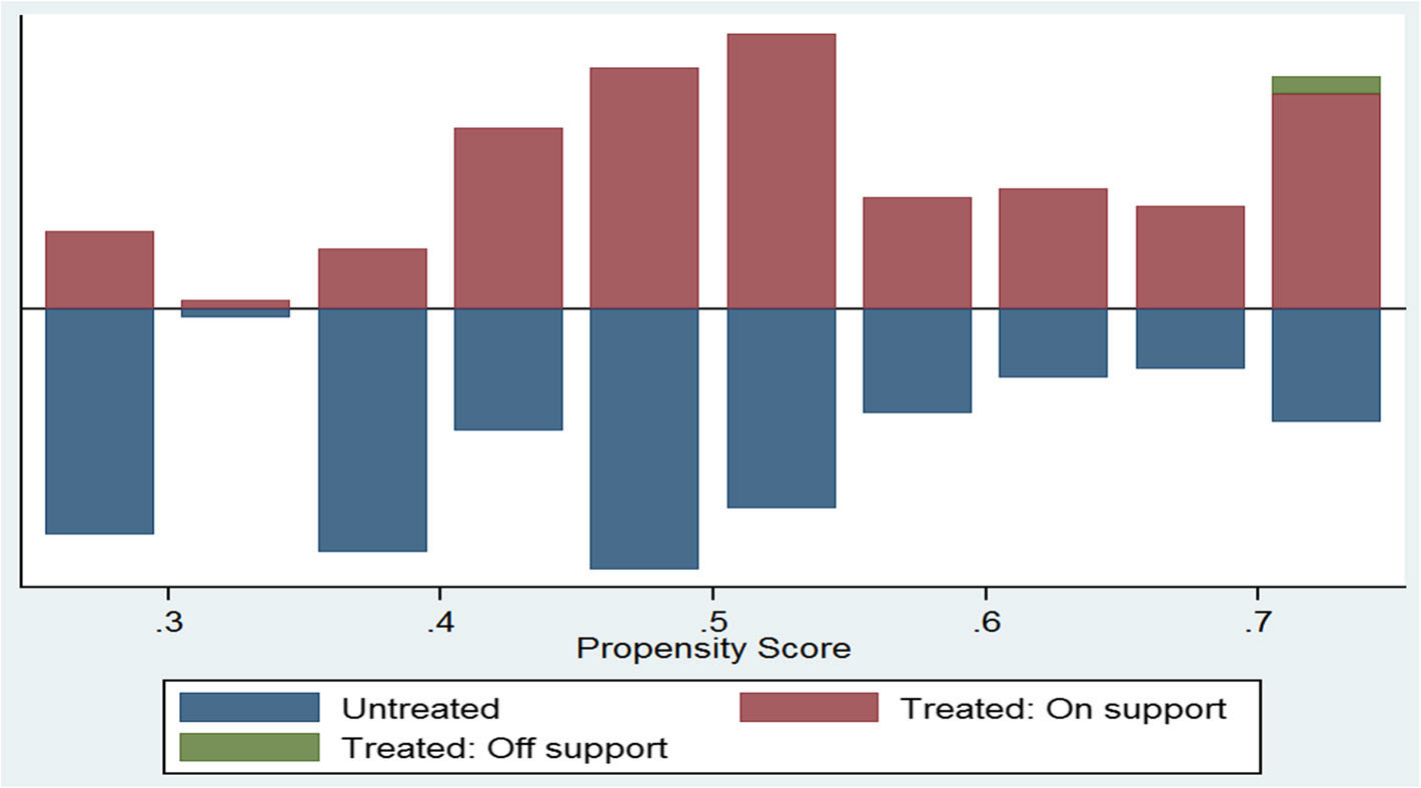
Distribution of propensity scores among intervention and control groups

Using the PSM analysis, we estimate the counterfactual (average treatment effect on the treated if they were not treated) after matching using the nearest neighbor approach. Based on the propensity scores generated after matching the intervention and the control groups, the two groups did not significantly differ with regards to key baseline sociodemographic variables such as sex of the child, marital status of caregiver and the household wealth index (Fig. 3). The average treatment effect after matching revealed the existence of a statistically significant effect of the intervention at the endline (ATT: -0.214; 95% CI: − 0.36, − 0.07), with the baseline estimate showing a non-significant effect (ATT: -0.035; 95% CI: − 0.16, 0.09) as shown in Table 6.

**Table 6:** Average treatment effect on the treated estimation (ATT) *after matching*

Time-point	Intervention (n)	Control (n)	ATT	SE	*t*	95% CI
Baseline	166	159	-0.035	0.061	−0.573	−0.16, 0.09
Endline	164	156	−0.214**	0.073	−2.943	−0.36, − 0.07

**p < 0.01

*SE* = Standard errors

### Sensitivity analysis of PSM estimate

We tested for the robustness of the PSM analysis in order to account for possible unmeasured confounder by conducting a sensitivity analysis using the *sensatt* command in Stata. The re-estimated effect (using ‘readwrite’ variable as the simulated confounder) both at baseline and endline suggested that the effect of the intervention on malaria prevalence was insensitive to the omission of plausible unobserved confounders [30] (Table 7).

**Table 7:** ATT estimation with simulated confounder

Time-point	ATT	SE	Outcome effect (OR)	Selection effect (OR)
Baseline	−0.033	0.077	1.050	0.806
Endline	−0.239	0.075	1.002	0.845

Both the outcome and the selection effect are odds ratios from logit estimations.

*SE* = Standard errors

### Difference-in-differences (DiD) model

We used the difference-in-difference model to account for the non-parallel modification before and after introducing the intervention as shown in Table 8. The baseline estimate revealed a non-significant difference (difference: − 0.066; *p* = 0.216) in the prevalence of malaria between the intervention and the control groups. On the other hand, the endline estimate revealed a statistically significant difference (difference: − 0.221; *p* < 0.001) between the two groups. The overall impact of the intervention revealed a statistically significant reduction of malaria prevalence (DiD: − 0.154; *p* = 0.043) among children whose caregivers received the intervention.

**Table 8:** Effect of the SMS intervention with DiD

	BASELINE			ENDLINE			Diff-in-Diff
Outcome	Control	Intervention	Diff (BL)	Control	Intervention	Difference (BL)	
Malaria	0.584	0.651	0.066	0.378	0.599	0.221***	0.154**
SE			0.054			0.054	0.076
t			1.24			4.09	2.03
P > │t│			0.216			< 0.001	0.043

***p < 0.01 **p < 0.05 SE = Standard Errors Diff (BL) = Difference at Baseline Diff (EL) = Difference at Endline

## Discussion

The findings of our study showed that sending theory-driven voice SMS via mobile phones to caregivers is an effective means of reducing the prevalence of malaria among children under-five. We found that the reduction of malaria prevalence among children under-five in the intervention group was higher compared with the control group. This is, to our knowledge, the first study that attempts to assess the effect of a theory-driven mHealth intervention on the prevalence of malaria among children under-five living in a resource-limited setting.

Evidence from previous studies suggests that mHealth interventions have the potential to improve the sub-optimal adherence of caregivers to artemisinin-based combination therapy [13], patient adherence rates for follow-up [31] and vaccination coverage [6]. However, the majority of these studies failed to highlight the effect of such intervention on health outcomes. In our study, malaria prevalence among children under-five was found to be higher than the national estimate of 53% [25] of children living in rural areas. This may be due to the absence of urbanization, a phenomenon identified as an important risk factor in other studies to have significant parasitological and clinical implications [32, 33].

We observed a higher reduction in the prevalence of malaria among children in the intervention group. This could be due to an improved health behaviors among the caregivers, an endpoint associated with adoption of healthy behaviors [34, 35]. As posited by the EPPM behavior change theory employed in our study, threat motivates action but perceived efficacy determines the nature of that action [28]. Thus, high perceptions of threat and efficacy result in an increase in adoption of positive behaviors [25, 29]. Based on the assumptions of the EPPM, the intervention group were less likely to be in a state of denial of recognizing malaria as a threat to their children and taking preventive action due to high perceptions of threat and efficacy. In our study, the intervention group had improved threat and efficacy perceptions, and therefore likely engaged in preventive behaviors, example, ensuring their children sleep consistently under a treated bed net. This explains the observed positive effect of the intervention on malaria prevalence among children in the intervention group. Other possible reasons for the observed effect could be due to the fact that there were significant differences in marital status of the caregivers and household wealth between the intervention and control group, although controlled in the analysis. In line with the hypothesis of our study, the treatment effect observed after matching key covariates suggests that receiving theory-driven health meassages via mobile phones could significantly reduce the likelihood of a child getting malaria. The observed counterfactual outcome further suggests that the fear appeal nature of the health messages likely caused a reduction in malaria prevalence, a situation which would not have been observed if caregivers in the intervention group had not received the intervention. Although limited evidence exists on the use of mHealth as a malaria control tool, findings from other studies that used SMS revealed a significant suppression of viral loads of HIV patients [36] and reduction in visible plaque scores among preschool children [37]. On the contrary, results of a randomized control trial [38] revealed no significant effect of SMS messages on pregnancy outcomes, a result attributed to the use of a small sample size. Nonetheless, in our study, the observed higher reduction in malara prevalence among children in the intervention group could be attributed to the content of the message, designed to emphasize the negative health implications of malaria and at the samt time provide preventive advice, which may have influenced the health practices of the caregivers. In line with this assertion, studies that employed this fear appeal tactic have reported a similar improvement in health outcomes [39, 40]. The observed improvements in these studies were attributed to an increase in the perceived threat and efficacy among the participants as posited by the behavior change theory [24, 29]. Nonetheless, these findings suggests that mHealth interventions could have a significant effect on health outcomes if the messages are theoretically constructed to influence the cognitive, normative and behavioral aspects of the target population [41]. However, in order to maximize the effectiveness of health messages on cognition about a health threat, prior information of the stage of change is paramount. In our study, we acknowledged the existence of an inherent fear of malaria among the caregivers, likely due to previous experience or prior knowledge of the disease, a factor documented to affect the effectiveness of the messages delivered to the target audience [42, 43]. The messages were therefore tailored to elicit positive health response from the caregivers, taking cognizance of the stage of behavior change.

### Limitations

This study had some limitations. Firstly, although we attempted to control as many threats as possible, the internal validity of the study design due to lack of random assignment of participants to either an intervention or a control group may have been affected. The matching analysis was, however, conducted to minimize the threat to this validity. Secondly, due to the poor data recording and limited resources, it is possible that not all the febrile cases that reported at the health facilities were confirmed by RDT or microscopy although recorded in the clinician’s report. Thirdly, children who were treated at home with anti-malarial drugs due to fever may not have exactly been confirmed cases of malaria. Also, maturation effect may have affected the outcome due to the frequency of the messages delivered and the duration of the study. This may be due to participants being tired of listening to the same pattern of messages for the entire duration of the study, as the rate at which participants answered and listened to the voice SMS was 15% lower than expected. In addition, the cluster effect and the confounding effects of other factors including social and behavioral change programs on malaria control organized in some communities was not adequately addressed in the analysis. Also, the reliability of the questionnaire used to measure the EPPM constructs may not reflect the true stage of the behavior change of participants due to the low Cronbach’s alpha. Finally, recall bias may have affected the interval validity of the estimate, although health records of majority of the children who were reported ill were assessed and confirmed to have had malaria at baseline and endline. These may have affected the observed effect, although the sensitivity analysis suggests otherwise.

## Conclusion

Findings of this study suggest that innovative approaches involving the use of mobile technology could be used as a strategy for controlling malaria in children under-five especially those living in rural settings where healthcare resources are limited. The Ghana National Malaria Control Program could adopt the use of mobile phones as part its malaria control strategies.

## Additional file

Additional file 1: SMS script used. (DOCX 19 kb)

## Data Availability

The data collected for the study which has been analysed and presented are available at the corresponding authors’ institution and is available upon formal request.

## Abbreviations

AEIR: Annual Entomological Inoculation Rates
ATT: Average Treatment Effect on the Treated
CHRPE: Committee for Human Research, Publications and Ethics
CIA: Conditional Independence Assumption
DiD: Difference-in-Differences
EPPM: Extended Parallel Process Model
GDHS: Ghana Demographic and Health Survey
GPS: Global Positioning System
HIV: human immunodeficiency virus
JHS: Junior High School
mHealth: Mobile health
mHIS: mobile phone-based health information system
MTN: Mobile Telephone Networks
PSM: Propensity Score Matching
RDT: Rapid Diagnostic Test
SMS: Short message service
WHO: World Health Organization
