# Supplementary material for: Mobile phone short message service (SMS) as a malaria control tool: a quasi-experimental study
Source: BMC Public Health. 2019 Aug 29;19:1193. doi: 10.1186/s12889-019-7336-6 (PMC6716929; doi:10.1186/s12889-019-7336-6)
Supplement: Supplementary file 1 — SMS script used. (DOCX 19 kb) [file 12889_2019_7336_MOESM1_ESM.docx]

**Additional file 1: SMS scipt used**

|  |  |  |  |
| --- | --- | --- | --- |
| **Message themes and delivery time, with two weeks delivery interval** | ***Knowledge about malaria*** | ***Threat***  ***(Severity and susceptibility)*** | ***Efficacy***  ***(Self-efficacy & Response efficacy)*** |
| ***Month 1***  *Set 1* | - Malaria is a life-threatening disease caused by parasites that are transmitted to people through the bites of infected mosquitoes. - Most deaths occur among children living in Africa where a child dies every minute from malaria. - The parasites are spread to people through the bites of infected Anopheles mosquitoes. | - Last year, malaria caused an thousands of deaths among African children. - If malaria is not treated within 24 hours, it can progress to severe illness often leading to death. | - Malaria is preventable and curable. Increased malaria prevention and control measures are dramatically reducing the malaria burden in many places. - You can prevent malaria and avert death among every child under 5 by  1. Ensuring that they sleep in ITNs everyday. 2. Indoor residual spraying 3. Use of mosquito coils and repellents. 4. Ridding your environment of stagnant waters |
| *Set 2* | - Malaria is an acute febrile illness. Symptoms of malaria include: fever, headache, chills and vomiting. - Children with severe malaria frequently develop one or more of the following symptoms: severe anaemia, respiratory distress and cerebral malaria. - Early diagnosis and treatment of malaria reduces disease and prevents deaths. Protect your family from malaria. | - Malaria can result in miscarriage and low birth weight, especially during first and second pregnancies. | Everybody in Ghana can do their part to prevent malaria in children by:   1. Ensuring that they sleep in ITNs everyday. 2. Indoor residual spraying 3. Use of mosquito coils and repellents. 4. Ridding your environment of stagnant waters |
| ***Set 3*** | - Malaria is caused by parasites that are transmitted to people through the bites of infected mosquitoes and easily kills. - Children living in Africa die every minute from malaria. - The parasites are spread to people through the bites of infected Anopheles mosquitoes. | - Every year thousands of African children die because of malaria. - If malaria is not treated within 24 hours, it can progress to severe illness, often leading to death. | - Malaria is preventable and curable. Increased malaria prevention and control measures are dramatically reducing the malaria burden in many places. - You can prevent malaria and avert death among every child under 5 by  1. Ensuring that they sleep in ITNs everyday. 2. Indoor residual spraying 3. Use of mosquito coils and repellents.  - Ridding your environment of stagnant waters |
| ***Set 4*** | - Malaria is a common childhood illness that kills a child every second. - Early symptoms of malaria can include irritability and drowsiness, with poor appetite and trouble sleeping. These symptoms are usually followed by chills, and then a fever with fast breathing | - . Last year, malaria caused an thousands of deaths among African children. - If malaria is not treated within 24 hours, it can progress to severe illness often leading to death. | - Malaria is preventable and curable. Increased malaria prevention and control measures are dramatically reducing the malaria burden in many places. - You can prevent malaria and avert death among every child under 5 by  1. Ensuring that they sleep in ITNs everyday. 2. Indoor residual spraying 3. Use of mosquito coils and repellents. 4. Ridding your environment of stagnant waters |
| *Set 5* | - Unfortunately, children under the age of five die easily of malaria and its associated complications. | - If your child has malaria, he/she needs immediate attention because it could lead to death. | You can help save a life by making children sleep under an LLIN every night. It is the cheapest and most effective way to prevent malaria.  Also, you can prevent malaria by indoor residual spraying, use of mosquito coils and repellents, and ridding your environment of stagnant waters. |
